# Utility of the Growth Differentiation Factor-15 in the Differential Diagnosis of Follicular-Patterned Lesions of the Thyroid on Cytopathologic and Histopathologic Samples

**DOI:** 10.7759/cureus.46206

**Published:** 2023-09-29

**Authors:** Prasanna V Perumal, Neelaiah Siddaraju, Sunil K Saxena, Soundravally Rajendiran, Ramachandra V Bhat

**Affiliations:** 1 Pathology, Jawaharlal Institute of Postgraduate Medical Education and Research (JIPMER), Puducherry, IND; 2 ENT, Jawaharlal Institute of Postgraduate Medical Education and Research (JIPMER), Puducherry, IND; 3 Biochemistry, Jawaharlal Institute of Postgraduate Medical Education and Research (JIPMER), Puducherry, IND; 4 Pathology, Indira Gandhi Medical College and Research Institute (IGMC & RI), Puducherry, IND

**Keywords:** fine-needle aspiration, the bethesda system, thyroid, gdf-15, follicular variant, papillary carcinoma, follicular hyperplasia, follicular carcinoma, follicular adenoma, follicular pattern

## Abstract

Background

Follicular-patterned lesions are a major gray zone in thyroid cytopathology. The recently introduced 2022 World Health Organization (WHO) classification emphasizes the importance of genetic alterations in thyroid neoplasms with the introduction of certain newer terminologies that are expected to cause remarkable changes in cytopathologic and histopathologic reporting. Although molecular assays such as the Afirma gene expression classifier and the ThyroSeq are already in use, there has been an ongoing search for further reliable molecular markers. The growth differentiation factor-15 (GDF-15) is one among them. This study aimed to determine the diagnostic utility of GDF-15 mRNA expression in frozen tissue and fine-needle aspiration (FNA) samples from follicular-patterned thyroid lesions and neoplasms.

Methodology

The real-time quantitative polymerase chain reaction was performed on 75 frozen tissue and FNA samples each from 19 cases of follicular thyroid hyperplasia (FTH), 10 nodular goiters (NGs), 17 follicular thyroid adenomas (FTAs), eight follicular thyroid carcinomas (FTCs), 12 follicular variant of papillary thyroid carcinomas (FVPTCs), and nine classic papillary thyroid carcinomas (CPTCs) that were diagnosed according to the 2017 WHO classification of thyroid neoplasms. The GDF-15 mRNA expression in all these cases was assessed and compared with the control thyroid tissue samples. One-way analysis of variance and the Kruskal-Wallis test were performed using GraphPad Prism 8 software to determine the significance of differences in the GDF-15 mRNA levels among various thyroid lesions.

Results

A higher GDF-15 mRNA expression was noted in the malignant thyroid neoplasms including FTC, FVPTC, and CPTC in comparison to FTA, with a fold change between the malignant and benign groups being more than 244.18 times. A difference in the fold change was noted between FTH and FTA with an increase in GDF-15 mRNA level in the latter, which was statistically not significant.

Conclusions

The fact that GDF-15 mRNA was studied both on fine-needle aspiration cytologic and the frozen tissue material and that the majority of the lesions studied were follicular-patterned establishes the GDF-15 as a potential marker not only for diagnosing malignant thyroid neoplasms of the follicular epithelium but also in distinguishing benign and malignant follicular-patterned neoplasms of the thyroid.

## Introduction

The prevalence of palpable thyroid nodules is estimated to be around 12.2% in India while the global incidence ranges between 5% and 15% [1,2]. Thyroid cancer constitutes 94.5% of all endocrine malignancies of which follicular thyroid carcinoma (FTC) accounts for 15% of all thyroid malignancies [3]. Despite certain limitations, fine-needle aspiration cytology (FNAC) is considered the best diagnostic tool available for the preoperative assessment of thyroid nodules [4]. The cytodiagnosis of most malignancies involving the thyroid including metastatic deposits is fairly straightforward, while diagnostic dilemmas are common with follicular-patterned lesions and neoplasms having a monomorphic cytomorphology. In the 2017 World Health Organization (WHO) classification of thyroid neoplasms, the differential diagnoses in such scenarios included the adenomatous nodule or follicular thyroid hyperplasia (FTH), follicular thyroid adenoma (FTA), FTC, and follicular variant of papillary thyroid carcinoma (FVPTC) [5], with the inclusion of the non-invasive follicular thyroid neoplasm with papillary-like nuclear features (NIFTP) described in 2016, further complicating the already existing cytodiagnostic complexities [6].

There have been notable changes with regard to follicular-patterned lesions in the 2022 WHO classification of thyroid neoplasms [7], which are yet to be addressed in the Bethesda system for reporting thyroid cytopathology [5]. In the current WHO classification, the infiltrative histologic type of follicular papillary thyroid carcinoma (FPTC), which in the 2017 WHO classification termed FVPTC is considered under the BRAF-like tumors, while other cytologic gray zone follicular lesions including NIFTP and encapsulated invasive follicular variant of papillary thyroid carcinoma (EIFVPTC) are referred to as RAS-like follicular tumors. The lesions earlier reported as FTH, adenomatous/colloid nodule, and multinodular or nodular goiter (MNG/NG) have all been grouped into a single entity called follicular nodular disease (FND). These modifications will have a considerable impact on the reporting of thyroid specimens in both histopathology and cytopathology. The emphasis laid on the established genetic alterations is one of the novel features of the 2022 WHO classification [7].

Constant efforts have been in progress for the last few decades to resolve the preoperative cytodiagnostic dilemmas associated with follicular-patterned thyroid lesions. Studies have evaluated the role of immunohistochemical (IHC) markers on FNA cell block preparations as well as on histopathologic specimens, with variable success and conflicting results [8]. The 2015 American Thyroid Association Management Guidelines recommend molecular tests for a preoperative cytologic distinction of benign and malignant thyroid nodules. Microarray techniques have led to the discovery of various molecular markers and assays applicable to cytologic and histologic samples [9-13].

In recent years, certain less-explored genes such as the growth differentiation factor-15 (GDF-15), cyclinD2 (CCND2), protein convertase-2 (PCSK2), trefoil factor-3 (TFF-3), adrenomedullin-3 (ADM-3) [14], and trophoblast cell‑surface antigen‑2 (TROP‑2) [15] have been emphasized as potential molecular markers in the neoplastic thyroid workup. The Afirma gene expression classifier (GEC), ThyroSeq and their improved versions, the gene sequencing classifier (GSC), and ThyroSeq v3 are the currently available molecular tools for the preoperative thyroid workup [16,17].

The GDF-15, belonging to the human transforming growth factor-β (TGF-β) family, has been studied in various malignancies including thyroid cancer. The GDF-15 gene is also known by several other names that reflect its varied functions in various neoplastic and non-neoplastic conditions. It is said to be involved in the activation of the STAT3 signaling and the stimulation of ERK phosphorylation, as well as the Smad signaling in thyroid cancer cells. It also has been claimed to play a crucial role in mitochondrial stress-induced thyroid cancer [18]. Some earlier studies have established its role in the Akt pathway and thyroid tumorigenesis including FTC [19].

The polymerase chain reaction (PCR) is an effective molecular tool with a relatively simple and practicable approach to quantifying gene expression. Many studies have used real-time quantitative polymerase chain reaction (RT-qPCR) for studying gene expression patterns in thyroid neoplasms [10,13,20-24]. The present study has assessed the quantitative mRNA expression of GDF-15 in resolving the diagnostic issues encountered in common follicular-patterned lesions.

## Materials and methods

The study was conducted in the Department of Pathology, Jawaharlal Institute of Postgraduate Medical Education and Research (JIPMER), Puducherry, India, in collaboration with the Departments of Surgery, Otorhinolaryngology and Biochemistry, JIPMER, Puducherry and the Department of Pathology, Indira Gandhi Medical College and Research Institute (IGMC & RI). The study was begun after obtaining permission from the Research Committees of JIPMER and IGMC & RI as well as the Ethics Committee of JIPMER. All well-circumscribed or encapsulated lesions with histopathologic diagnoses of the adenomatoid nodule/FTH, FTA, FTC, and FVPTC that had the preoperative TBSRTC interpretations/diagnoses of benign: NG, adenomatous nodule/FTH, atypia of undetermined significance/follicular lesion of undetermined significance (AUS/FLUS), follicular neoplasm/suspicious for FN (FN/SFN), or a specific diagnosis of FVPTC (per the 2017 classification) were included in the study, while those without a preoperative FNAC/interpretations/diagnoses were excluded. The sample size was estimated based on the area under the curve of 0.725 in the receiver operating characteristic (ROC) curve with a negative: positive ratio of 0.666 (derived from our previous hospital records) and with an alpha error of 0.05 and a beta error of 0.20 using the Medcalc. 15.6.1 Software. Thus, a total of 75 thyroid nodules with cytohistopathological correlation including 19 FTHs, 10 NGs, 17 FTAs, eight FTCs, 12 FVPTCs, and nine classic papillary thyroid carcinomas (CPTCs) were studied. In all these cases, the adjacent normal thyroid tissue obtained from the postoperative thyroidectomy specimens served as the control. Informed consent from the patients was obtained for their participation in the study before the pre-therapeutic FNA procedure and before surgery.

Specimen collection for mRNA extraction

Immediately after the surgery, the FNA material was collected from the lesional and control areas of the postsurgical thyroid specimens, followed by tissue samples from the corresponding lesional and control areas; thus, four samples from each case were included in the study. The samples were transferred immediately in liquid nitrogen and stored in a deep freezer at -80°C. The RNA was obtained and purified using a High Pure RNA Isolation kit (Roche Life Science) from stored frozen specimens (0.2 g) and FNAC samples. Further, the isolated RNA was treated with DNase.

The quality and quantity of RNA were measured using a Nanodrop ultraviolet (UV) spectrophotometer (ND-2000, Thermoscientific, USA). The RNA-isolated samples with Nanodrop readings of 1.9 or above at 260/280 nm wavelength were subjected to the cDNA conversion process. After confirming a comparable quality of RNA isolates from FNAC and frozen tissue samples, 500 ng of RNA from the individual samples was subjected to the cDNA conversion process using a prior quality-checked, readymade kit containing the reverse transcriptase enzyme (Roche Life Science) in a reaction volume of 20 µL. The final quantification of the converted cDNA was done using the spectrophotometer.

The RT-qPCR was performed using UPL probe number 50 (Roche Life Science) for the GDF-15 gene and the study primers were as follows: forward primer - GCAAGAACTCAGGACGGTGA and reverse primer - TGGAGTCTTCGGAGTGCAAC. The β-actin (probe Number-9) served as the housekeeping gene (positive control). The RT-qPCR was performed in triplicates with cDNA generated from one FNAC and two frozen tissue samples from the lesional area of each surgical specimen. The average CT value of the test sample, determined by the mean of the triplicate-run CT values, was considered the final test CT value. Similarly, the average CT value of the control tissue was derived through RT-qPCR. The normalization of the sample was done using the adjacent normal thyroid tissue. The delta CT (∆CT) value of each of the test and control samples was determined by subtracting the CT value of the test and the control from the CT value of the housekeeping gene CT. The delta delta CT value (∆∆CT), i.e., the relative expression of the GDF-15 gene was determined by the Schmittgen and Livak method after normalizing the test sample by subtracting the test ∆CT from the control ∆CT. Finally, the two-power negative delta delta CT(2-∆∆CT) was computed to determine the fold change. One-way analysis of variance and the Kruskal-Wallis test within the GraphPad Prism software (version 8.0) were used for statistical analysis. The non-parametric two-tailed tests were used, and a p-value of <0.05 was considered statistically significant.

## Results

The patient’s age ranged from 19 years to 75 years, with a median of 56 years. There were 60 females and 15 males with a male-to-female ratio of 1:4. The preoperative cytologic diagnoses/interpretations per TBSRTC and their histopathologic correlation are provided in Table 1.

**Table 1:** Cytohistopathologic correlation of cases included in the study. TBSRTC: the Bethesda system of reporting thyroid cytopathology; AUS: atypia of undetermined significance; FLUS: follicular lesion of undetermined significance; FN: follicular neoplasm; SFN: suspicious for follicular neoplasm; SM: suspicious of Malignancy; FTH: follicular thyroid hyperplasia; NG: nodular goiter; FTA: follicular thyroid adenoma; FVPTC: follicular variant of papillary thyroid carcinoma; FTC: follicular thyroid carcinoma; HTA: Hürthle cell adenoma; CPTC: classic papillary thyroid carcinoma

Cytologic diagnoses (number of cases)	Histopathological diagnoses (number of cases)
TBSRTC-I (non-diagnostic): 1	FTH (1)
TBSRTC-II (benign): 37	NG (10); FTH (16); FTA (9); and FVPTC (2)
TBSRTC-III (AUS/FLUS): 6	FTH (2) and FTA (4)
TBSRTC-IV (FN/SFN): 11	FTC (6); FTA (1); HTA (2); and FVPTC (2)
TBSRTC-V (SM): 12	FVPTC (5); CPTC (5); FTC (1); and FTA (1)
TBSRTC-VI (malignant): 8	CPTC (4); FVPTC (3); and FTC (1)
Total: 75	Total: 75

**Figure 1:**
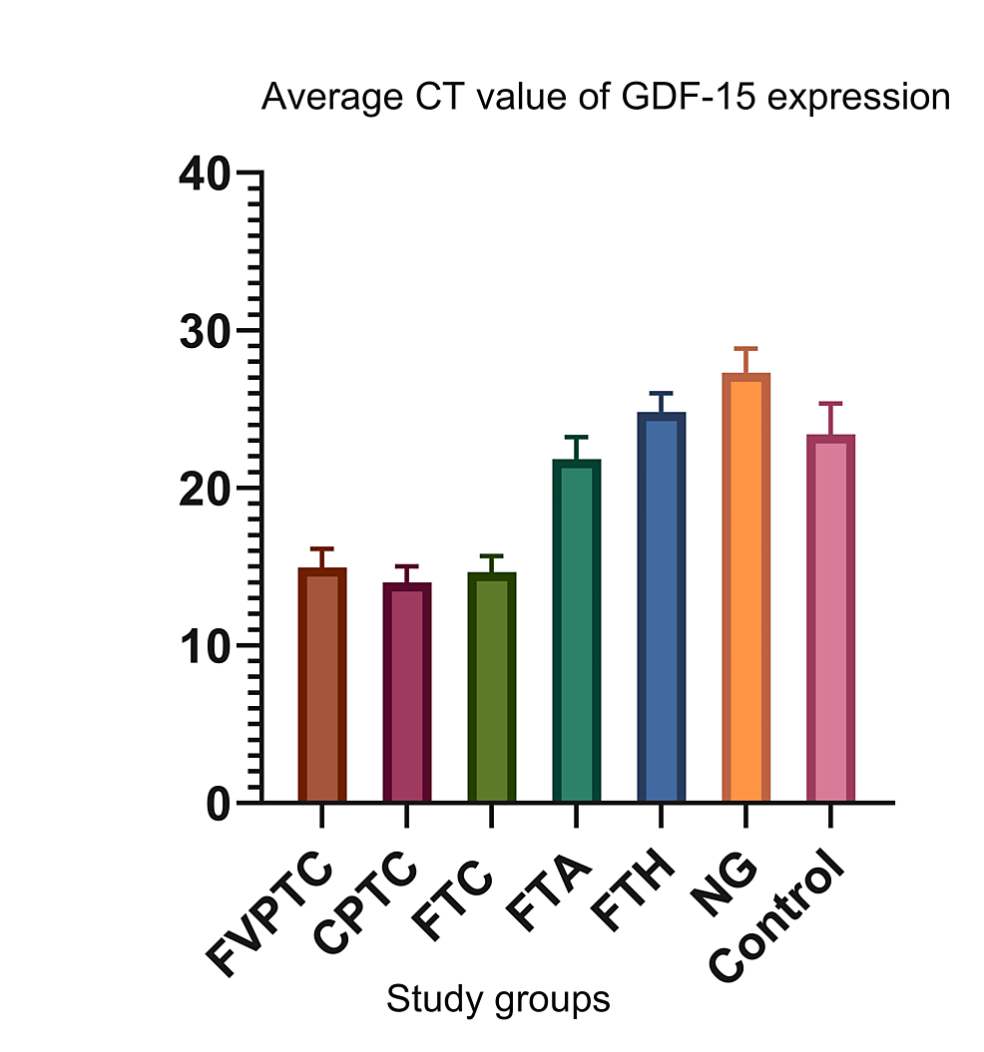
The mean and standard deviation of the GDF-15 mRNA expression in various study groups. GDF-15: growth differentiation factor; CT: cycle threshold; FVPTC: follicular variant of papillary thyroid cancer; CPTC: classic papillary thyroid cancer; FTC: follicular thyroid carcinoma; FTA: follicular thyroid adenoma; FTH: follicular thyroid hyperplasia; NG: nodular goiter

The mRNA expression of the GDF-15 was demonstrated in all frozen tissue samples and FNAs. Following the final histopathologic diagnosis (gold standard), the study samples were grouped according to the nature of the thyroid lesion. The mean and standard deviation of the CT values of the GDF-15 mRNA expression of the various study groups are shown in Figure 1.

The post hoc power was calculated on OpenEpi software version 3 using the benign fold change mean value of 4.24 and standard deviation of 2.37 along with the malignant fold change mean value of 244.18 and standard deviation of 217.54 with a 95% confidence interval showed the power of more than 99%. The GDF-15 mRNA expression was found to be statistically significant in distinguishing FTC vs. NG/FTH. The variation in GDF-15 expression was found to be useful in differentiating FTC vs. FTA, with FTCs showing consistently elevated GDF-15 mRNA levels. The GDF-15 mRNA expression was more significantly upregulated in the FTC group compared to the FTA, FTH, and NG groups, with p-values of 0.0077, <0.0001, and <0.0001, respectively. Similar to the FTC group, the GDF-15 mRNA was also increased in the CPTC (p < 0.01) and FVPTC (p < 0.01) groups (Table 2).

**Table 2:** The GDF-15 mRNA expression in various thyroid tumors. GDF-15: growth differentiation factor; NG: nodular goiter; FTH: follicular thyroid hyperplasia; FTA: follicular thyroid adenoma; FTC: follicular thyroid carcinoma; FVPTC: follicular variant of papillary thyroid carcinoma; CPTC: classic papillary thyroid carcinoma; *: significant; NS: non-significant

Multiple comparisons between groups	Mean rank difference between the groups	P-value	Summary
NG vs. FTH	-11.45	>0.9999	NS
NG vs. FTA	-21.53	0.1136	NS
FTH vs. FTA	-10.08	>0.9999	NS
NG vs. FTC	-55.12	<0.0001	****
NG vs. FVPTC	-43.78	<0.0001	****
NG vs. CPTC	-49.31	<0.0001	****
FTH vs. FTC	-43.66	<0.0001	****
FTH vs. FVPTC	-32.33	0.0015	**
FTH vs. CPTC	-37.86	0.0005	***
FTA vs. FTC	-33.58	0.0077	**
FTA vs. FVPTC	22.25	0.0392	*
FTA vs. CPTC	22.78	0.0428	*
FTC vs. FVPTC	-11.33	>0.9999	NS
FTC vs. CPTC	-5.806	>0.9999	NS
FVPTC vs. CPTC	-5.528	>0.9999	NS

**Figure 2:**
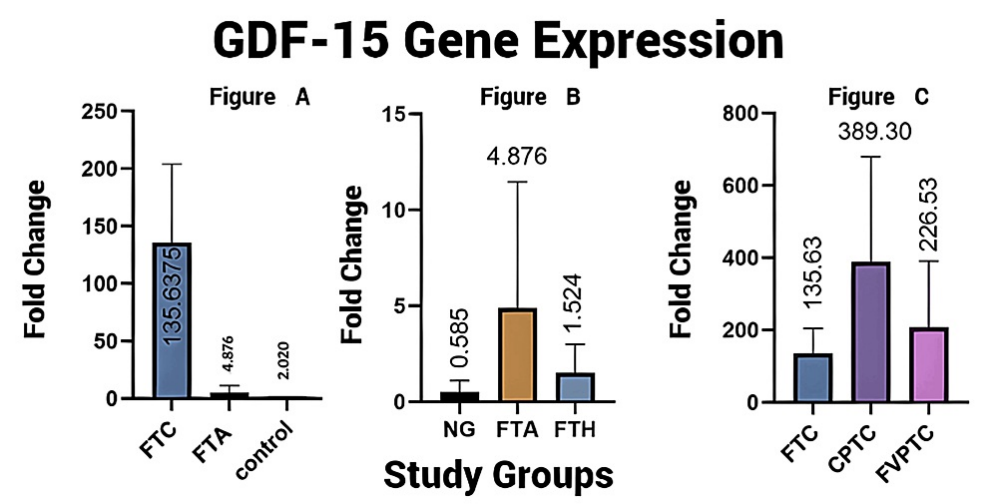
(A) Difference in the relative GDF-15 mRNA expression between FTC and FTA with normal control. (B) The relative GDF-15 mRNA expression in the FTA, FTH, and NG groups. (C) The relative GDF-15 mRNA expression in the FTC, CPTC, and FVPTC groups. GDF-15: growth differentiation factor; FTC: follicular thyroid carcinoma; FTA: follicular thyroid adenoma; NG: nodular goiter; FTH: follicular thyroid hyperplasia; CPTC: classic papillary thyroid cancer; FVPTC: follicular variant of papillary thyroid cancer Note: A one-fold increase in mRNA expression in the control tissue compared to a lower baseline level in NG is possibly an insignificant physiologic variation.

However, there was no statistical difference in the elevated GDF-15 mRNA levels between FTC and FVPTC. Thus, the GDF-15mRNA expression pattern noted in our study was useful in differentiating benign vs. malignant thyroid follicular lesions, though it failed to distinguish between FTC and FVPTC. The variation in the fold change between FTC and FTA was glaring with FTC having more than 130 times a fold change than FTA with an average fold change of 135.6 times. FTA showed relative expression with an average of 4.8 times increased fold change. An average of 1.5 times fold change of the GDF-15 relative expression was noted in FTH, while no significant expression was noted in NG. Between FTH and FTA, a relatively higher fold change was noted in FTA with a mean of 4.8 times higher upregulation despite which GDF-15 failed to differentiate FTA vs. FTH and FTA vs. NG. A significant upregulation of GDF-15 mRNA expression was observed in the CPTC and FVPTC groups. An average increase in fold change of 389 times was noted in CPTC, while it was 226.5 times for FVPTC (Figures 2A-2C).

Our data strongly suggested the possibility of an aberrant GDF-15 expression occurring in all malignant thyroid tumors included in the study, with the expression being strikingly higher in CPTC and FVPTC than in FTC, though statistically not of much help in differentiating among them. The GDF-15 expression was exceedingly higher in malignant compared to the benign tumors and the control tissue with a significant fold change between FTC and FTA. Thus, the consistency and efficacy of our results were established on both cytologic and histopathologic samples with a successful quantification of GDF-15 mRNA using the RT-qPCR technique.

## Discussion

Despite the practical issues associated with the distinction between benign and malignant nodules, the FNA technique remains an irreplaceable preoperative tool for the evaluation of suspected thyroid nodules [5]. The 2022 WHO classification of thyroid neoplasms with its newer terminologies and the emphasis on genetic variations stresses the need for refinement in the neoplastic thyroid workup [7]. Currently, TBSRTC has been accepted as the standard pattern of reporting. The third edition of TBSRTC has already been in circulation with only minimal changes from the second edition [5]. However, significant changes made in the current WHO classification are expected to bring substantial alterations in the subsequent TBSRTC editions. In the 2022 WHO classification, follicular cell-derived thyroid neoplasms are categorized into three families or classes including benign, low-risk, and malignant neoplasms. The terms FND and differentiated high-grade thyroid carcinoma are introduced to account for multifocal hyperplastic/neoplastic lesions and differentiated thyroid carcinomas with high-grade features, respectively. With the term Hürthle cells replaced by oncocytes, the Hürthle cell thyroid adenoma (HTA) and Hürthle cell thyroid carcinoma (HTC) are redesignated as oncocytic thyroid adenoma (OTA) and oncocytic thyroid carcinoma (OTC), respectively. The encapsulated invasive follicular variant of PTC (EIFVPTC) is redefined as a distinct tumor type with a RAS-like genetic profile, while the infiltrative follicular variant of PTC (FPTC) is now considered a histologic subtype of PTC with a BRAF-like genetic profile, while the term variant is used to describe genetic alterations, as in cases of EIFVPTC which we did not come across in the present study. We also did not come across other low-risk and high-risk malignancies of the follicular epithelium including NIFTP.

The cases included in the present study were cytologically reported per the second edition of TBSRTC. Of our 75 cases with histopathologic confirmation, 66 were follicular-patterned lesions comprising NG, adenomatoid nodule/FTH, FTA, HTA, FTC, and FVPTC (Table 1). Of these, the first two entities are together referred to as FND in the current WHO classification, and the terminologies FTA and FTC have remained unaltered. HTA is replaced by OTA. Our cases of FVPTC can be redesignated as FPTC [7].

Looking at the cytohistopathologic discrepancy noted among our follicular-patterned thyroid lesions, one can easily grasp the gravity of the interpretative dilemmas faced by cytopathologists. Two FVPTCs reported as NGs (false negative) were attributable to a sampling error, with histopathology revealing features of nodular goiter in the tissue adjacent to the tumor, while clinical and cytologic evidence of bony metastasis led to a definitive diagnosis of FTC (TBSRTC-VI) on thyroid FNA in one of our FTCs. There were two OTAs, while no cases of OTC were reported.

The criticality of an accurate preoperative diagnosis for the surgical management of thyroid nodules led to the advent of various ancillary studies including molecular techniques such as ThyroSeq and the Afirma GEC assays [8,16,17]. The ThyroSeq v2 version was designed to detect mutations in >1,000 hotspots of 14 thyroid cancer-related genes (AKT1, BRAF, CTNNB1, GNAS, HRAS, KRAS, NRAS, PIK3CA, PTEN, RET, TP53, TSHR, TERT, and EIF1AX) and for 42 types of gene fusions or rearrangements known to occur in thyroid cancer (RET, PPARG, NTRK1, NTRK3, BRAF, and ALK), while the Afirma GEC uses a microarray measurement of mRNA of 167 genes that includes driver genes such as HRAS, NRAS, KRAS, and BRAF-V600E along with p53 and others. A meta-analysis by Borowczyk et al. documented a pooled sensitivity and specificity of 84% (74-91%) and 78% (50-92%), respectively, for ThyroSeqv2, while it was 98% (96-99%) and 12% (8-20%), respectively, for Afirma GEC. The analysis revealed pooled positive predictive value and negative predictive value of 58% (31-81%), and 93% (89-97%), respectively, for ThyroSeq v2 and 45% (37-53%) and 91% (85-96%), respectively, for GEC. According to the meta-analysis, the ThyroSec v2 had a better specificity, while the Afirma GEC had a better sensitivity [16].

The most recent version, the ThyroSeq v3, is a 112 gene DNA- and RNA-based targeted next-generation sequencing (NGS) assay that tests for five categories of genetic alterations including point mutations, indels, gene fusions (GFs), copy number alterations (CANs), and the gene expression alterations (GEAs). The GEA studies are performed by comparing mRNA expressions via NGS, detected in an ultrasound-guided thyroid FNA against a panel of 167 genes identical to the Afirma GEC. Of these 167 genes, 142 are involved in the algorithm that identifies benign gene expression patterns, while the other 25 genes are involved in filtering out rare neoplasms and assessing for BRAF-V600 mutations [17].

The GDF-15, also referred to as macrophage inhibitory cytokine-1, non-steroidal anti-inflammatory drug-inducible gene-1, placental transforming growth factor-β, prostate-derived factor, and placental bone morphogenetic protein, is a gene with diverse physiologic and pathologic functions [12,18,25]. It is upregulated by certain stress-related proteins such as interleukin (IL)-1, tumor necrosis factor-α, IL-2, macrophage stimulating factor-1, and p53. A high level of GDF-15 is expressed in the normal placenta and prostate, wherein it is regulated by androgens and calcitriol; however, it can be induced in other cell types such as cardiomyocytes, adipocytes, macrophages, endothelial, and vascular smooth muscle cells. The GDF-15 has been shown to signal through the glial cell-derived neurotrophic factor (GDNF) family receptor α-like (GFRAL), which is solely expressed in the human brain stem and is associated with GDF-15-mediated anorexia and cachexia. However, the fact that pregnancy which is also known to have elevated GDF-15 levels but not associated with anorexia and cachexia perhaps indicates a likelihood of GFRAL-independent GDF-15 action in conditions such as tissue injury, inflammation, metabolic disease, immune tolerance, and cancer [26]. Among malignancies, GDF-15 has been shown to be strongly expressed in prostatic, pancreatic, colorectal, gastric, urothelial, and renal carcinomas as well as melanoma, and at relatively lower levels, in cervical, breast, endometrial, thyroid, and pancreatic carcinomas [12,18,26]. While among thyroid cancers, most studies emphasized its role in PTC [18], rare studies have more specifically dealt with its role in thyroid follicular neoplasms [12,14]. Being a member of the TGF-β family, it is also involved in the regulation of Smad pathways. Some studies have reported a frequent overexpression of Smad-4 and Smad-7 in thyroid cancer [27].

In our study, the GDF-15 mRNA expression was well-correlated with the diagnosis of follicular-patterned thyroid malignancies, viz., FTC and FVPTC. It was also strongly expressed in CPTC which served as a positive control. Our GDF-15 mRNA results were quite useful in differentiating FTA and FTC. Only two studies in the literature have evaluated the role of GDF-15 in distinguishing benign and malignant follicular-patterned thyroid nodules. The GDF-15 was one of the eight genes analyzed by the RT-qPCR method in the study by Krause et al. [14]. In this study, both the neoplastic and the adjacent normal tissues from the thyroidectomy specimens were studied in 52 benign and 46 malignant thyroid lesions. Of the 46 malignant lesions, 20 each were FTCs and CPTCs, while six were undifferentiated carcinomas. Substantial upregulation of GDF-15 was noted when it was used in combination with TFF3, CCND2, HGD1, and thyroglobulin (TG) genes with 1,469, 32.67, 130.24, and 302 times the fold change, respectively, for distinguishing FTA vs. FTC. Even on a single gene basis, the increased GDF-15 expression was found to be statistically significant. In the study by Weber et al., the GDF-15 was validated in combination with protein convertase-2 (PCSK2) and CCND2 genes. The snap-frozen thyroid tissues from 12 FTCs, five FTAs, seven adenomatous nodules, and two normal thyroids were used for performing RT-qPCR. Striking differences were noted between FTA and FTC, with a fold change of 10.2 times and 263 times, respectively, for CCND2 and PCSK2, both of which exhibited significant downregulation. The GDF-15 revealed fold changes that were 5.5 times upregulated. Thus, the three-gene profile had a sensitivity of 100%, specificity of 94.7%, and diagnostic accuracy of 96.7% [12]. It is noteworthy that some of our FTAs and FTHs showed an upregulated GDF-15 mRNA, although not to the extent of overlapping with malignant follicular-patterned neoplasms. It needs to be explored if this observation could reflect an ongoing mutational change in histologically benign follicular nodules [7].

Neither Krause et al. nor Weber et al. studied the GDF-15 protein expression by IHC. The GDF-15 IHC findings of the follicular-patterned thyroid lesions of our study have been documented before, wherein the majority of the malignant thyroid lesions including FTC, FVPTC, and CPTC were found to be positive. However, unlike the unambiguously increased GDF-15 mRNA levels observed in the malignant follicular-patterned lesions, some of our benign lesions also revealed a GDF-15 positivity on IHC, which limits its practical application at least as a single IHC marker [28].

Though the literature addresses the role of GDF-15 in thyroid tumorigenesis and progression, further studies are essential to clearly understand its precise mechanisms in thyroid oncology. Nevertheless, consistently elevated GDF-15 mRNA levels in all our follicular-patterned thyroid malignancies (FTC and FVPTC) matching with that of CPTC strongly support its role as a potential marker for diagnosing thyroid malignancy of follicular epithelium. Preoperatively, the cytomorphologic features do play a role in the distinction of FTC, FPTC, and PTC. Features such as papillary pattern, frequent intranuclear inclusions, prominent grooving, and other well-established criteria are helpful in diagnosing CPTC, while FTC and FPTC are distinguished by the presence or absence of the subtle cytomorphologic details such as the enlarged ovoid nuclei, elongated follicles, scanty gummy colloid, and other established but less commonly observed features [5,29].

Currently, the GDF-15 is not employed in any of the available molecular assays and we believe that its inclusion in the ThyroSeq v3 as well as Afirma GEC and other upcoming molecular assays could improve their efficacy as preoperative molecular diagnostic tools, which is crucial for the surgical management of the thyroid nodules.

Study limitations

Despite the sample calculation performed before executing the study, the numbers of the varied follicular-patterned lesions were too low with no cases of NIFTP and EIFVPTC encountered in the study. As our study was a pure research project, the FNA material was procured from the postoperative thyroidectomy specimens which resulted in better cell yield with the extraction of larger amounts of RNA responsible for the highly significant results documented by us. Hence, further studies are essential to know if similar results could be obtained with the material procured through non-guided or ultrasound-guided FNA to establish its practical utility.

## Conclusions

The demonstration of GDF-15 mRNA by RT-qPCR technique with the FNA and frozen tissue material procured from the postoperative thyroidectomy specimens of the pathologic thyroid nodules and the consistently elevated GDF-15 mRNA levels in all malignant thyroid nodules including FTC, FVPTC, and CPTC strongly support the role of GDF-15 as a potent molecular marker for distinguishing benign vs. malignant thyroid nodules, in general, and benign vs. malignant follicular-patterned lesions, in particular. Based on our results, we strongly believe that the incorporation of GDF-15 in the gene panel of the newer versions of the Afirma GEC and ThyroSeq assays could enhance their sensitivity, specificity, and predictive values.
